# 

**Published:** 1984-07

**Authors:** 


### BRISTOL MEDICO-CHIRURGICAL JOURNAL

JULY 1984

VOLUME 99 (iii)

NUMBER 371

